# Vortex ring behavior provides the epigenetic blueprint for the human heart

**DOI:** 10.1038/srep22021

**Published:** 2016-02-26

**Authors:** Per M. Arvidsson, Sándor J. Kovács, Johannes Töger, Rasmus Borgquist, Einar Heiberg, Marcus Carlsson, Håkan Arheden

**Affiliations:** 1 Lund University, Department of Clinical Sciences Lund, Clinical Physiology, Skane University Hospital, Lund, Sweden; 2 Cardiovascular Biophysics Laboratory, Cardiovascular Division, Washington University School of Medicine, Box 8086, 660 St Euclid Avenue, St. Louis, MO 63110 USA; 3 Dept. of Cardiology, Arrhythmia Clinic, Lund University Hospital, Lund University, 22185 Lund, Sweden; 4 Dept. of Biomedical Engineering, Faculty of Engineering, Lund University, 221 85 Lund, Sweden

## Abstract

The laws of fluid dynamics govern vortex ring formation and precede cardiac development by billions of years, suggesting that diastolic vortex ring formation is instrumental in defining the shape of the heart. Using novel and validated magnetic resonance imaging measurements, we show that the healthy left ventricle moves in tandem with the expanding vortex ring, indicating that cardiac form and function is epigenetically optimized to accommodate vortex ring formation for volume pumping. Healthy hearts demonstrate a strong coupling between vortex and cardiac volumes (R^2^ = 0.83), but this optimized phenotype is lost in heart failure, suggesting restoration of normal vortex ring dynamics as a new, and possibly important consideration for individualized heart failure treatment. Vortex ring volume was unrelated to early rapid filling (E-wave) velocity in patients and controls. Characteristics of vortex-wall interaction provide unique physiologic and mechanistic information about cardiac diastolic function that may be applied to guide the design and implantation of prosthetic valves, and have potential clinical utility as therapeutic targets for tailored medicine or measures of cardiac health.

What determines the shape of the heart? Since the early days of the universe, fluid motion conforms to the laws of fluid dynamics. These laws constitute a strong guiding force for biological evolution, and flow-function adaptations are plentiful in nature, for example the energy-saving vortex slalom of trout^1^, the jet propulsion of squid^2^, the maneuverability of jellyfish^3^, and the occasional leisurely vortex ring play of dolphins^4^. Intracardiac flow is known to influence cardiac embryonic development through transduction of endothelial shear forces; alteration of flow patterns bring about macroscopic changes in cardiac geometry by means of epigenetic modifications^5^,^6^,^7^,^8^,^9^. However, the “blueprint” for cardiogenesis, i.e., the hemodynamic determinant of final cardiac shape, remains unknown. Vortex ring formation is a prominent feature of intracardiac flow^10^,^11^,^12^,^13^, that is known to differ between healthy and failing hearts^14^,^15^. In idealized water tank experiments, vortex ring formation is chiefly influenced by the ratio between the pulsatile inflow volume and the dimensions of the inlet, the vortex formation ratio (VFR, also known as *L/D* [length/diameter], stroke ratio, or vortex formation time)^2^,^14^,^16^,^17^,^18^,^19^. The possibility of using VFR as a marker of cardiac filling performance has recently been under debate^20^,^21^,^22^,^23^. Given consistent inflow conditions, vortex ring formation is a stable and predictable phenomenon^17^,^18^, and vortex rings have the potential to transport fluids while minimizing energy loss^24^, which is likely to gain importance during physical stress when the heart rate increases^25^. Vortex rings in the heart therefore provide a recurring fluid structure with a consistent amount of shear along its outer boundary, which could potentially constitute a hemodynamic framework, or “blueprint” for ventricular adaptation. For efficient filling, the ventricle must both recoil to aspirate atrial blood (Doppler E-wave) and simultaneously be large enough to fully accommodate the vortex ring it generates. In contrast large, failing ventricles are poor suction pumps, generating vortices smaller than normal and having high wall tension (due to Laplace’s law). Hypothetically then, for volume pumping in diastole the optimal ventricle is the smallest possible that will match endocardial expansion to the expanding boundary of the simultaneously generated vortex ring (Fig. 1).

To investigate the spatiotemporal behavior of the vortex ring, we have developed and validated the first *in vivo* method for determining its three-dimensional outer boundary^14^,^26^, through post-processing of four-dimensional magnetic resonance imaging flow measurements. The method uses Lagrangian coherent structures (LCS) which by definition show the boundary layers present between mixing fluids of different origins. For the non-expert reader, this is exemplified by a naturally occurring LCS found between merging flows in the Amazon River (Fig. 2a). The brown and black waters remain separated by LCS for several kilometers; particle trajectories will by definition never cross an LCS surface. In the heart, LCS arise between inflowing blood (demarcating the outer limits of the vortex ring) and the residual blood that remains inside the ventricle after ejection^14^. LCS thus visualize the history of the flow; portions of blood separated by LCS have different origin when traced backwards in time. LCS analysis differs from other vortex ring analyses in that it visualizes the outer borders of the vortex ring, not the vortex cores or local vorticity, and as such constitutes a fundamentally new approach to intracardiac vortex rings that may shed light on the problem of vortex-wall interaction.

We hypothesized that to optimize filling, the growth of the vortex ring is closely matched to the motion of the endocardial surface of the ventricular wall. Pathological enlargement of the heart was hypothesized to disrupt this optimized flow-function relationship between vortex and wall. Our aim was therefore to quantify the dynamics of the vortex ring boundary in relation to the left ventricular endocardium throughout diastole.

## Results

**Figure 2:**
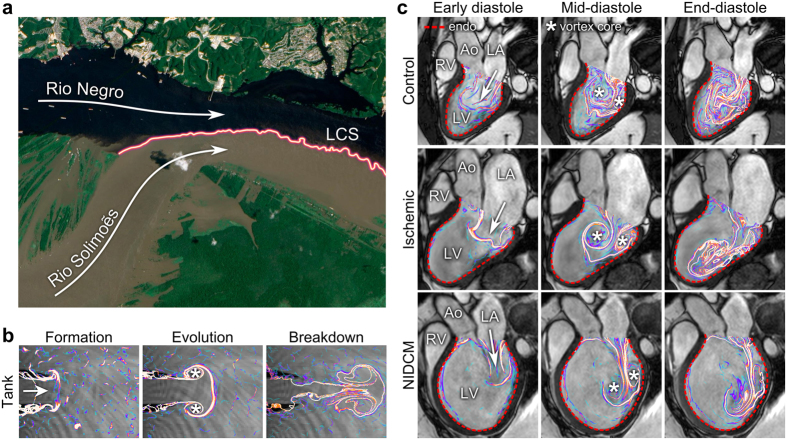
Flow patterns by Lagrangian Coherent Structures. (**a**) The black water of the Rio Negro meets the muddy water of the Rio Solimoẽs outside Manaus, Brazil. The border separating flows of different origins (colored line) is, by definition, a Lagrangian Coherent Structure (LCS). The LCS persists for several kilometers and grows increasingly uneven and complex as eddies contribute to mixing of the two flows. Image modified from an original by NASA Earth Observatory, with permission^42^. (**b**) Water tank experiment with LCS analysis of vortex rings. As water is injected from a nozzle into a tank, a symmetrical vortex ring forms (left), detaches and evolves (middle), then gradually breaks down into more complex flow patterns (right). (**c**) LCS showing vortex ring formation during filling of the LV. Healthy control (top), patient with ischemic cardiomyopathy (middle), and non-ischemic dilated cardiomyopathy (bottom). After the formative phase, the vortex ring evolved close to the endocardial border (red dotted line) in the control, gaining additional complexity compared to the water tank model. In the patients, vortex ring formation occurred without an obvious connection to the endocardium, and the vortex ring moved along the inferior LV wall towards the apex. LV, left ventricle; RV, right ventricle; LA, left atrium; Ao, aorta; *, vortex core.

**Figure 3:**
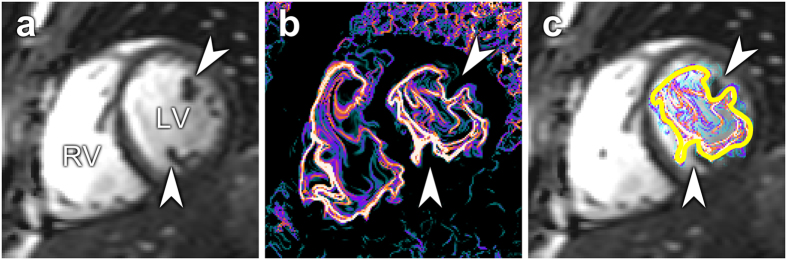
(**a**) Short-axis midventricular slice showing cardiac anatomy. Arrowheads indicate the position of papillary muscles. (**b**) Corresponding LCS image. The vortex ring adapts to intracavitary structures, causing an irregular appearance. (**c**) LCS superimposed over anatomy. The yellow line shows the manual delineations of the vortex ring boundary. LV, left ventricle; RV, right ventricle.

**Figure 4:**
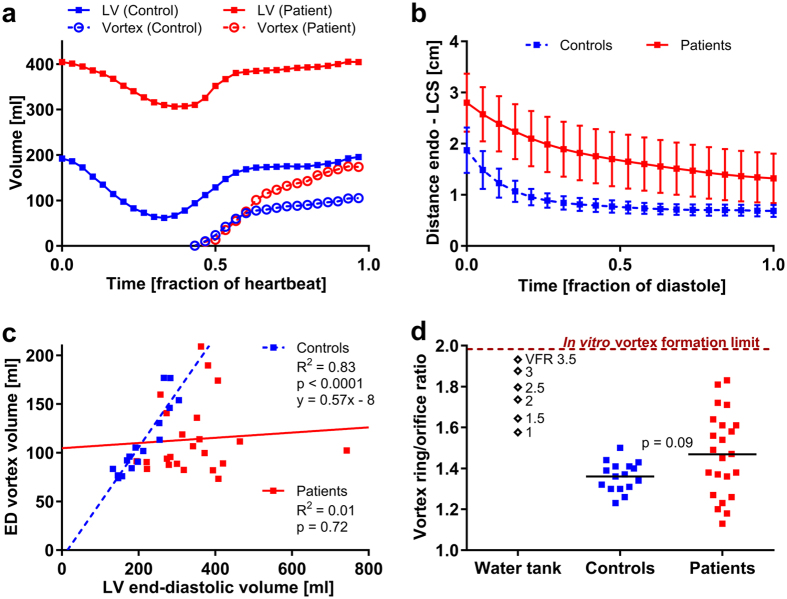
Measurements of vortex ring parameters. (**a**) Volume-time curves of one control and one patient. Vortex ring development was initially similar between patients and controls. In the patient, however, the vortex ring continued to grow independently of LV volume, leading to additional entrainment of ventricular blood. (**b**) Distance between vortex ring surface (LCS) and endocardium of the left ventricle (mean ± SD). In controls, the vortex ring boundary was in close proximity to the endocardium after the initial growth phase. The dilated ventricle displayed continuous evolution of the vortex ring throughout diastole. (**c**) Vortex ring volume at end-diastole was linearly correlated to LV end-diastolic volume in controls, and filled on average 53% of the ventricle. This implies that LV chamber size and motion are tuned to accommodate the vortex ring in healthy hearts regardless of total heart size. Conversely, patients demonstrate a loss of optimized vortex-wall interaction. (**d**) The ratio between peak vortex ring diameter and inlet orifice diameter in controls and patients was lower in controls, a consequence of the elongated shape of the vortex ring at rest. The ratios in patients were more scattered, consistent with lower remaining filling reserve.

We studied 16 healthy controls and 23 heart failure patients with pathological dilatation of the left ventricle (characteristics shown in Tables 1 and 2). We also performed phantom experiments in a custom-built vortex ring generator tank to study how vortex ring formation occurs in the absence of constraining boundaries. These water tank experiments have been validated and described in greater detail in^26^. Figure 2b shows the evolution of an axisymmetric vortex ring in the tank (see also Supplementary video 1 and Supplementary Fig. S1). The evolution of the vortex ring in a healthy control and two patients is shown in Fig. 2c (Supplementary videos 2 and 3). Vortex ring formation *in vivo* demonstrated additional complexity compared to the water tank model, due to interaction with intracavitary structures such as papillary muscles (Fig. 3). In controls, the vortex ring grew slightly after the early filling phase due to entrainment of ventricular blood (Fig. 4a). By end-diastole (after atrial contraction), the vortex ring contained on average 53% (95% CI: 49–58%) of the total LV volume. After early rapid filling and onward, the average distance between vortex boundary and LV wall was remarkably consistent in all controls, as opposed to patients (Fig. 4b). Vortex ring volume at end-diastole was strongly correlated to LV end-diastolic volume (Fig. 4c) but the ratio between vortex ring and ventricular size was unaffected by inflow velocity (Table 3).

In contrast, the enlarged, failing heart displayed a marked spatiotemporal discrepancy between vortex boundary and endocardium. The vortex boundary formed at a greater distance from the endocardium, and gradually decreased the distance to the endocardium throughout diastole (Fig. 4b). By end diastole, the vortex ring filled on average 35% of the ventricle (95% CI: 30–41%) and was not correlated to the total LV volume (Fig. 4c).

### Vortex ring behavior: water tank vs heart

Our experiments on vortex rings in a water tank replicated the previous finding that vortex ring size is strongly dependent on the ratio between stroke volume and inlet size^2^,^17^,^18^. This vortex formation ratio (VFR) characterizes the amount of fluid that can be incorporated into a vortex ring without generating an energy-wasting trailing jet; in water tank experiments, conditions with VFR above ~4 produce turbulent jets^19^. Using pump settings with VFR = 3.5, near the upper limit for vortex growth, the vortex ring diameter grew to 1.93 times the diameter of the inlet nozzle in the tank (Fig. 4d). In controls, however, the vortex assumed a more elongated shape, and the ratio between vortex cross-sectional diameter and mitral valve diameter was lower compared to the water tank (Fig. 4d). In patients, the ratio between vortex ring diameter and mitral annular diameter more resembled the conditions in the water tank, indicating that vortex-wall interaction was disturbed and that a larger portion of the vortex formation reserve capacity was utilized at rest in some subjects compared to controls. Mitral valve area did not differ between controls and patients (p = 0.31).

## Discussion

This study has demonstrated a spatiotemporal coupling between the left ventricular vortex ring and endocardial wall during diastole, implying that the healthy heart is tuned to accommodate vortex ring formation. Intracardiac vortex ring formation provides a stable, recurring, and predictable flow phenomenon that may dictate and optimize cardiogenesis by providing endocardial shear forces. Failing, dilated hearts show dissociation between the vortex ring and endocardium, indicating the potential utility of vortex ring-derived metrics for quantification of cardiac health.

The effects of altered flow or cardiac loading conditions on cardiac development was investigated by Hove *et al.*^5^ and Sedmera *et al.*^6^, although the experimental setups did not enable the authors to distinguish which particular flow pattern was the driving force. We propose that the vortex ring is the hemodynamic blueprint for cardiac shape, and that disruption of vortex ring formation leads to altered shear along the endocardial border. Blood flow is known to influence endothelial cell structure and alignment through membranous mechanotransducers^7^,^8^,^9^, which provides a mechanistic explanation for how vortex rings may influence ventricular dimensions by shear forces, activating cellular remodeling/growth programs until the ventricle conforms to the vortex dimensions. Indeed, congenital mitral valvular stenosis has been associated with abnormally small LVs^27^. Altering inflow conditions by ligation of the mitral valve annulus during cardiogenesis would enable validation of the proposed causal connection between inflow parameters and ventricular dimensions.

Vortex formation in humans is spatially more complex compared to water tank experiments^14^, as shown in Figs 2 and 3. The proximity to the endocardial walls means that the vortex ring will interact with, and be deformed by ventricular structures. Both the asymmetry of the mitral valve apparatus, and the presence of papillary muscles add to the lopsided and somewhat irregular appearance of the intracardiac vortex ring, complicating direct comparison with tank models^20^,^28^. These limitations notwithstanding, we found a very consistent ratio between the largest vortex ring diameter and mitral valve diameter in controls, but large differences between controls and some patients; and both groups showed discrepancies compared to the water tank experiments. Mitral valve area was similar between patients and controls, so an explanation for this discrepancy must reside elsewhere. It appears that the vortex ring of the healthy heart is deformed by its close proximity to the wall, so that maximum diameter is not attained and vortex ring growth is limited by ventricular dimensions, unlike in the dilated heart where the vortex ring can continue to grow throughout diastole, even after termination of the E-wave (Fig. 4a). At rest, therefore, the mitral valve is not fully utilized for vortex ring generation in the healthy heart. Instead, there may be a “reserve capacity” for vortex ring formation, which would allow for sustained energy-efficient mass transfer during demand for higher cardiac output. Given the close coupling between vortex ring border and left ventricular wall, the only way to significantly increase the diameter of the vortex ring during exercise is to increase the radius of the left ventricle – a phenomenon previously observed using both echocardiography^29^ and MRI^30^. Such an arrangement keeps wall tension low during extended periods of rest, while preserving the capability to quickly increase cardiac output when necessary.

Cardiac remodeling is typically triggered during periods of stress, such as physical exertion or a pathological increase in cardiac load. Therefore, vortex-wall interaction during exercise is of potential interest for remodeling mechanics. Conversely, the ratio between vortex ring and mitral annular diameter may be a key factor in cardiac suction-pump efficiency during exercise; if the vortex formation capacity is nearly fully utilized at rest (high ratio), LV filling is likely to be a limiting factor for exercise tolerance. Hence the fluid dynamics of the failing heart resides closer to the limit for how large a vortex can be formed. We predict that the ratio in healthy hearts will increase during vigorous exercise, reflecting the optimized relationship between dynamic valve and flow properties and chamber size.

Vortex-wall interaction may be an important consideration for the long-term performance of prosthetic valves; if the implanted valve is too small in relation to the LV or its valve leaflet orientation alters inflow direction relative to normal, it may substantially impair optimal vortex generation. Therefore, the opportunity exists for prosthetic valve implants to be tuned to the dimensions of the left ventricle in the exercise state, to ensure optimal vortex ring formation under conditions where it matters most.

The narrow physiological range of vortex ring size relative to chamber size (Fig. 4c) implies this ratio has potential to serve as an index of diastolic function. Currently, 4D PC-MR is the only imaging modality that allows for accurate LCS computations of the complex flow field within the human left ventricle. LCS are inherently insensitive to image noise, and developments in graphical processing unit technology are rapidly shrinking computational times, increasing clinical availability. Although echo-determined VFR now includes diastolic chamber properties^22^, future studies should investigate whether vortex-ventricle coupling is further quantifiable by readily available clinical tools, e.g. transthoracic ultrasound with microbubbles^15^ or fully noninvasive Doppler technique^31^, and whether new indexes can augment or replace current clinical diastolic function parameters. For echocardiographic studies of vortex ring LCS, a two-dimensional approach may render significantly different results with small variations in placement of the acoustic beam due to the irregular appearance of the vortex ring, and these inconsistencies should be meticulously evaluated. For example, assuming a linearly tapering vortex ring, as recently presented^32^, is not consistent with our observations. Detailed study of the spatial localization of the vortex ring in relation to the endocardium may hold further information than given here, especially in patients with pathologically altered ventricular geometry.

From an evolutionary standpoint, matching endocardial wall movement to vortex growth implies optimization of the suction pump attribute of the LV. Because maintenance of cardiac output is a prime evolutionary directive for survival, the optimization of LV size and motion to accommodate the largest possible vortex is accompanied by numerous biological advantages. Vortex formation converts and preserves linear momentum as angular momentum^13^,^33^ and thereby prevents fluid kinetic energy from being converted into increased pressure^24^. Energy-wise, an optimal vortex is therefore one that contains all the inflowing blood without formation of a trailing jet. In terms of energy expenditures, systolic stroke work far exceeds blood flow, so vortex energetics are likely of small importance for global systolic function^34^. However, it has recently been shown that the kinetic energy of the diastolic vortex ring is significantly altered in heart failure^35^, implying that altered vortex ring dynamics may be a factor in the development of diastolic dysfunction and elevated filling pressures. A large vortex ring relative to the chamber size will also distribute inflow energy throughout the entire ventricle, which minimizes endocardial stasis of blood and thereby prevents thrombus formation. In contrast, a small vortex ring relative to chamber endocardial dimensions will interact less with the ventricular blood, provide a lower degree of mixing of the blood and hence increase the risk for thrombosis. Thus, any rinsing effect is likely to be diminished in hearts with vortex-wall dissociation. In most patients, the vortex ring moved along the inferior and lateral wall, typically dissolving near the apex. This flow pattern has been connected to increased risk for thrombus formation^10^, as the anterior wall is not exposed to fast-moving blood.

In conclusion, we have shown that the spatiotemporal behavior of the healthy left ventricle is optimized to accommodate the formation of an evolving diastolic vortex ring. Diastolic vortex ring features and dynamics are consistent across a wide size range of healthy hearts but significantly disturbed in heart failure, which increases our understanding of how fluid dynamics is coupled to, and governs cardiac shape and function. Vortex ring parameters carry implications for exercise physiology, cardiac surgery and design and implantation of prosthetic valves. Future studies should investigate vortex ring dynamics during exercise, and examine the clinical utility of vortex ring-derived metrics. The vortex ring behavior of the right ventricle also merits further characterization^16^,^24^,^36^.

## Methods

### Study population

The study was approved by the Regional Ethical Review Board in Lund, Sweden, and was performed in accordance with the Helsinki declaration. All subjects provided written informed consent. Imaging and population data are available upon written request to the communicating author.

16 healthy volunteers (11 male, average age 26) and 23 patients (19 male, average age 68) were enrolled in the study (see Table 1). Controls had normal ECG, no medications, and no history of cardiovascular or systemic disease. Inclusion criteria for patients were as follows: NYHA class I–IV heart failure, echocardiographic systolic ejection fraction <35%, and optimal pharmaceutical treatment (Table 2). Exclusion criteria for patients were: heart rate >100 beats per minute at cardiovascular magnetic resonance (CMR) examination, poor CMR image quality, severely reduced kidney function (GFR <30 ml/kg/min), chronic atrial fibrillation, unstable angina or recent myocardial infarction, severe valvular disease, and pregnancy.

### Imaging protocol

All subjects underwent CMR imaging using a 1.5 T Philips Achieva (10 normals and 21 patients) or a 3T Philips Achieva (6 normals and 2 patients), with a standardized protocol including functional imaging, and three-dimensional, three-component, time-resolved phase contrast (4D PC-MR) flow imaging. Patients additionally underwent late gadolinium enhancement imaging for infarct quantification.

We acquired balanced steady-state free precession (bSSFP) cine images in 2-chamber, 3-chamber, 4-chamber and short-axis views. Typical imaging parameters for bSSFP imaging at were: T_E_/T_R_ 1.4/2.8 ms and α 60° (1.5 T) or T_E_/T_R_ 1.8/3.7 ms and α 45° (3 T), in-plane spatial resolution 1.3 × 1.3 mm, slice thickness 8 mm, no interslice gap, and acquired temporal resolution 30 ms.

We also acquired validated 4D PC-MR flow images from a box covering the entire heart^37^,^38^. We used the following typical imaging parameters for the 4D PC-MR sequence on both scanners: T_E_/T_R_ 3.1–3.7/5.1–6.3 ms and α 8°, SENSE factor 2, spatial resolution 3 mm isotropic, segmentation factor 2, acquired temporal resolution 50 ms, and retrospective reconstruction to 40 timephases. We used a navigator pre-pulse for respiratory gating in 13 controls. 3 controls and all patients were scanned during free breathing.

Late gadolinium enhancement imaging was performed on all patients to determine infarct size. We used a validated protocol in current clinical use, consisting of ECG-triggered 2D phase sensitive inversion recovery and 3D inversion recovery gradient echo experiments. Images were acquired 10–20 minutes after intravenous administration of 0.2 mmol/kg gadolinium-based contrast agent (Dotarem, Guerbet, Roissy, France). Inversion time was chosen for best nulling of the myocardium. Infarct size was measured using the Segment Weighted automatic method with manual corrections where necessary, and calculated as infarct % of total myocardial mass^39^.

### Image analysis and LCS computation

Images were analyzed using a plugin for Segment 1.9 (Medviso, Lund, Sweden)^40^. A first-order polynomial fit to stationary tissue was used to compensate for phase background errors. Velocity aliasing was corrected using semiautomatic phase unwrapping. Spatial co-registration of bSSFP and 4DPC images was performed manually.

Time-resolved LCS were computed from 4DPC data in long-axis and short-axis views as previously described in greater detail^14^. Computations for LCS were performed on Graphical Processing Unit cards in the CUDA-C environment, resulting in an order of magnitude shorter computational times compared to the approach used in the previous study. Briefly, the 4DPC velocity data was linearly upsampled in time and space. In this velocity field, rectangular grids of particles spaced 0.8 mm apart were positioned according to the long-axis and short-axis views. Using a fourth-order Runge-Kutta method with time step 5 ms, the flow map was then computed for each point in the grid from each timeframe backwards in time to the beginning of vortex ring formation. The finite-time Lyapunov exponent (FTLE) was then computed and normalized to the 95^th^ percentile of FTLE values in the images in each time phase, and lines with FTLE values >50% of this value were considered as LCS. In cases where LCS ended blindly, delineations were connected with a straight line to the next available LCS in the vortex ring.

Left ventricular volumes were determined manual delineation of the endocardial border in short-axis bSSFP images. LCS were manually delineated in the short-axis view for each timeframe in diastole and were then imported and superimposed onto the bSSFP images. In cases where the LCS grew across the endocardium (due to image noise or intravoxel dephasing), LCS delineations were cropped to the endocardial delineations. The volume for each slice was calculated as the delineated area multiplied with the slice thickness. Slice volumes were then summed to produce a total LV and vortex ring volume for each timeframe.

### Quantification of vortex-wall interaction

To achieve a reproducible and robust measure of the average distance between endocardium and LCS, we took the following steps for each point in time: first, find the radius of a sphere whose volume is equal to the LV volume; second, find the radius of a sphere with a volume equal to the volume inside the LCS delineations; third, subtract the LCS sphere radius from the LV sphere radius.

To investigate the relationship between mitral valve dimensions and vortex ring size, we took the following steps: first, we reconstructed through-plane (2D) velocity maps for each slice in the short-axis view using the 4D data. Second, a region of interest (ROI) was drawn around mitral valve flow in the 2D velocity map. Mitral annular area was measured in the timeframe with peak flow, in the slice with the smallest flow profile area (Fig. S2). We then calculated the average mitral annular diameter by finding the diameter of a circle with area equal to the measured mitral valve opening. The same approach was used to find the largest LCS diameter at any time within the LV, which allowed for the computation of the ratio between largest LCS and mitral valve area.

### Diastolic function

To assess diastolic function in patients, we used: E/A, E/E′, mean E′, peak left atrial volume normalized to BSA, mitral E-wave deceleration time (MDT), E-wave peak velocity, and pulmonary venous flow profile, using normal values as referenced^41^. Flow profiles were extracted from the 4D flow data (Fig. S2), and E′ was measured in bSSFP 4-chamber view. Left atrial volume was delineated in the short-axis view.

### Statistical methods

We used GraphPad Prism 6 (GraphPad Software, La Jolla, USA) for all statistical analysis. Population demographics were compared using the Mann-Whitney-Wilcoxon test, with statistical significance assigned at p < 0.05. Linear correlation analysis was performed to investigate vortex and physiological parameters. For Table 3, we employed Bonferroni correction to compensate for 32 analyses, which rendered significance at p < 0.0015625.

## Additional Information

**How to cite this article**: Arvidsson, P. M. *et al.* Vortex ring behavior provides the epigenetic blueprint for the human heart. *Sci. Rep.* **6**, 22021; doi: 10.1038/srep22021 (2016).

## Supplementary Material

Supplementary Information

Supplementary Information

Supplementary Information

Supplementary Information

## Figures and Tables

**Figure 1:**
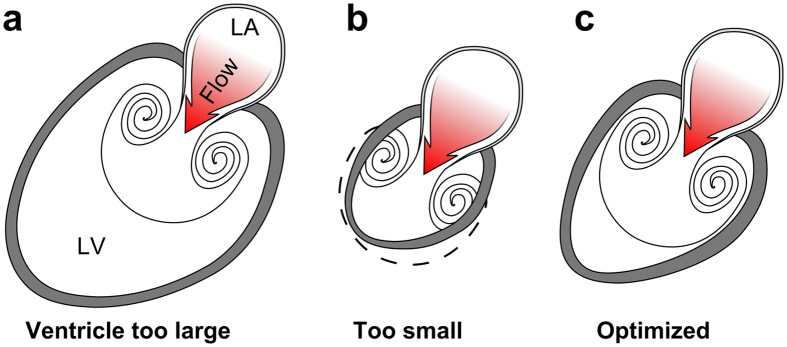
Possible anatomical arrangements for intracardiac vortex ring generation. (**a**) Vortex rings are formed by pulsatile flow through an aperture, such as flow from the left atrium (LA) to the LV through the mitral orifice. A large left ventricle can accommodate the full size of the vortex ring it generates, but the vortex cannot rinse the distant ventricular wall and prevent mural thrombus formation. Furthermore, large ventricles are poor suction pumps and have high wall tension which causes energy waste. (**b**) If the chamber is too small it cannot accommodate full vortex ring formation, and is therefore incompatible with efficient filling. (**c**) An optimized left ventricle enables energy-efficient filling by matching endocardial expansion to vortex ring expansion and formation, thereby facilitating rinsing at low pressure, and maintenance of low wall tension.

**Table 1:** Population demographics.

	Controls (*n* = 16)	Patients (*n* = 23)
Age, yrs	26 (22–31)	69 (49–87)****
Gender	11 M, 5 F	19 M, 4 F
Weight, kg	79 (49–95)	83 (59–117)
Height, cm	178 (155–198)	177 (159–192)
Body surface area, kg/m^2^	1.98 (1.45–2.29)	2.00 (1.63–2.44)
Heart rate, bpm	60 (38–77)	63 (51–88)
Systolic blood pressure, mmHg^†^	120 (95–150)	133 (91–170)
Diastolic blood pressure, mmHg^†^	70 (60–80)	75 (59–110)*
End-diastolic volume, ml	195 (133–305)	342 (195–743)****
End-systolic volume, ml	72 (53–143)	243 (124–665)****
Stroke volume, ml	125 (77–188)	85 (49–143)***
Ejection fraction, %	61 (53–69)	25 (10–39)****
LV mass, g	109 (68–241)	193 (96–292)***
LV sphericity index, end-diastole	0.38 (0.31–0.52)	0.58 (0.43–0.85)****
LV sphericity index, end-systole	0.30 (0.21–0.39)	0.55 (0.35–0.79)****

Median values (range). *p < 0.05, ***p < 0.001, ****p < 0.0001. ^†^Blood pressure readings were not available in three controls and one patient. Diastolic blood pressure could not be measured in one patient.

**Table 2:** Patient characteristics.

	Ischemic cardiomyopathy *n* = 12	Dilated cardiomyopathy *n* = 11
NYHA class*	I (2), II (6), III (3), IV (1)	II (3), III (6), IV (1)
Diastolic dysfunction (28)	Impaired relaxation = 5 Pseudonormal = 3 Restrictive = 4	Impaired relaxation = 4 Pseudonormal = 2 Restrictive = 2 Uncertain = 3
Scar size, % of LVM	17 (2–34)	0
ECG QRS duration, ms	162 (138–184)	167 (114–186)
Diabetes mellitus, *n*	1	1
Beta blocker, *n*	11	10
ACEi or ARB^†^, *n*	12	11
Diuretics, *n*	9	8
Platelet inhibitor, *n*	7	6
Lipid-lowering drug, *n*	8	5

^*^NYHA class was unknown in one DCM patient. ^†^ACEi, angiotensin converting enzyme inhibitor; ARB, angiotensin II receptor blocker.

**Table 3:** Linear correlation between physiological and vortex ring parameters.

	End-diastolic vortex volume		End-diastolic VV%	
	Controls	Patients	Controls	Patients
Body surface area	p = 0.034	p = 0.083	p = 0.33	p = 0.060
Age	p = 0.016	p = 0.30	p = 0.34	p = 0.64
Heart rate	p = 0.11	p = 0.24	p = 0.73	p = 0.074
End-diastolic volume	p < 0.0001 R^2^ = 0.83	p = 0.68	p = 0.78	p = 0.0068
Stroke volume	p < 0.0001 R^2^ = 0.76	p < 0.0001 R^2^ = 0.55	p = 0.75	p = 0.085
Ejection fraction	p = 0.70	p = 0.024	p = 0.90	p < 0.0001 R^2^ = 0.6
Peak filling rate	p = 0.0002 R^2^ = 0.65	p = 0.0003 R^2^ = 0.47	p = 0.82	p = 0.13
E-wave peak flow velocity	p = 0.17	p = 0.038	p = 0.66	p = 0.22

We applied Bonferroni correction to compensate for multiple analyses, and assigned significance at p < 0.0015625. R^2^ values are given for significant correlations. VV%, vortex volume as percentage of LV volume.
