# Supplementary material for: Vortex ring behavior provides the epigenetic blueprint for the human heart
Source: Sci Rep. 2016 Feb 26;6:22021. doi: 10.1038/srep22021 (PMC4768103; doi:10.1038/srep22021)
Supplement: Supplementary Information [file srep22021-s1.doc]

# Vortex ring behavior provides the epigenetic blueprint for the human heart

## Authors

Per M Arvidsson^a^, Sándor J Kovács^b^, Johannes Töger^a^, Rasmus Borgquist^c^, Einar Heiberg^a,d^, Marcus Carlsson^a^, Håkan Arheden^a*^

## Author affiliations

^a^Dept. of Clinical Physiology, Lund University Hospital, Lund University, 22185 Lund, Sweden

^b^Cardiovascular Biophysics Laboratory, Cardiovascular Division, Washington University School of Medicine, Box 8086, 660 St Euclid Avenue, St. Louis, MO 63110 USA

^c^Dept. of Cardiology, Arrhythmia Clinic, Lund University Hospital, Lund University, 22185 Lund, Sweden

^d^Dept. of Biomedical Engineering, Faculty of Engineering, Lund University, 221 85 Lund, Sweden

## Corresponding author

^*^Håkan Arheden ([hakan.arheden@med.lu.se](mailto:hakan.arheden@med.lu.se)), Department of Clinical Physiology, Lund University Hospital, 22185 Lund. Telephone: +46 46 173 328

## Supplementary information

###
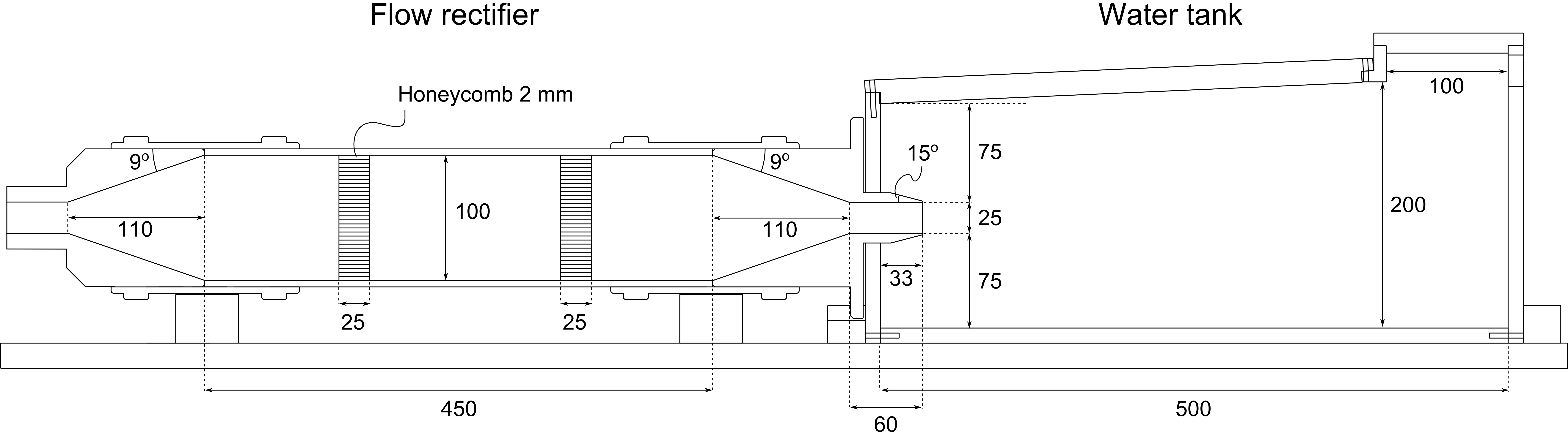


### Figure S1

Design of the vortex ring tank used to generate symmetrical vortex rings without interference from surrounding structures. The tank has previously been used for validation of the LCS method (26). The setup consists of a flow rectifier to minimize turbulence, and a water tank with a 25 mm nozzle similar to those used in earlier experiments (17,18). The vortex ring generator is driven by a programmable pump that allows for vortex ring generation at VFR 1-3.5.

###
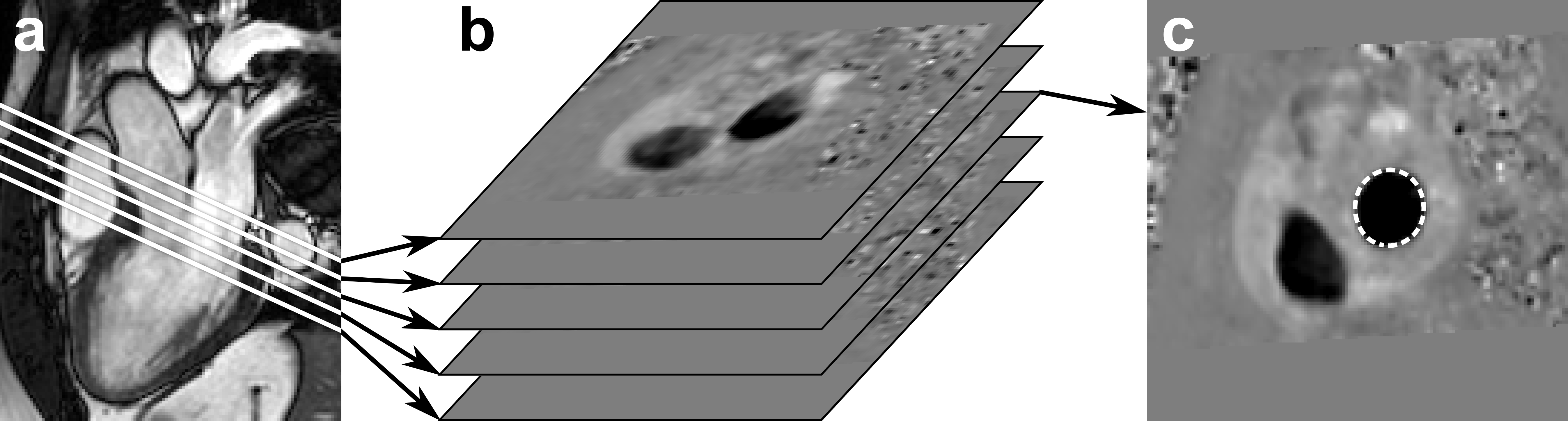


### Figure S2

Mitral valve orifice area was determined using phase-contrast images reconstructed from the 4D PC-MR dataset. **a**, Slices were perpendicular to long-axis images. **b**, Through-plane flow velocity image stack was reconstructed from 4D PC-MR data. The image with the smallest cross-sectional flow area at peak early rapid filling was selected to represent mitral valve orifice. **c**, A region of interest (white dotted line) identified mitral valve flow area (enclosed black segment).

###
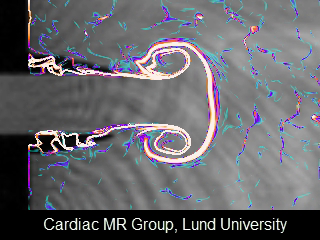


### Video 1

Symmetric vortex ring formation in the water tank, visualized using LCS. Water is injected from the nozzle on the left. The outermost LCS represents the outer boundary of the vortex ring.

###
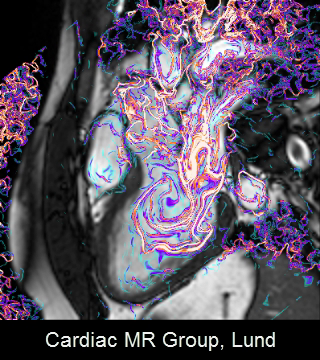


### Video 2

Visualization of vortex ring formation in the left ventricle of a healthy subject. LCS are generated from the onset of diastole. At the end of the movie, the endocardial border is shown by a red line, and the green line represents the end-diastolic vortex ring boundary. Note the close proximity between the two.

###
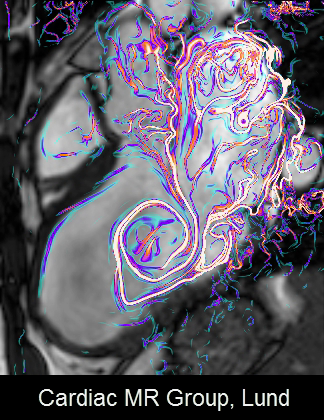


### Video 3

Visualization of diastolic vortex ring formation in a patient with ischemic cardiomyopathy. The distance between vortex ring (green line) and endocardial border (red line) is notably larger than in the healthy subject.
